# CBCT Assessment of the Anatomical Characteristics of Gubernacular Canal in Impacted Teeth

**DOI:** 10.30476/DENTJODS.2021.92400.1638

**Published:** 2023-03

**Authors:** Mahdiye Rayisi, Kianoosh Malekzadeh, Masoomeh Afsa

**Affiliations:** 1 Dept. of Maxillofacial Radiology, Faculty of Dentistry, Hormozgan University of Medical Sciences, Bandar Abbas, Iran; 2 Hormozgan Health Institute, Hormozgan University of Medical Sciences, Bandar Abbas, Iran

**Keywords:** Tooth Eruption, Tooth Impaction Impacted Tooth, Eruption Pathway Cone Beam Computed Tomography, Gubernacular Canal, Dental Follicle

## Abstract

**Statement of the Problem:**

Gubernacular canal (GC) is a canal that extends from the follicle of unerupted permanent teeth to the alveolar bone crest filled with remnants of the dental lamina. This canal is thought to guide tooth eruption and be related to some pathologic conditions.

**Purposes:**

This study aimed to determine the presence of GC and its anatomical characteristics in teeth, which failed to erupt normally on cone beam computed tomography (CBCT) images.

**Materials and Method:**

This cross-sectional study evaluated CBCT images of 77 impacted permanent and supernumerary teeth obtained from 29 females and 21 males. The frequency of GC detection, its location in relation to the crown and root, the anatomical surface of the tooth from which the canal has originated, and the adjacent cortical table to which the canal opens, along with the length of the GC were studied.

**Results:**

GC was observed in 53.2% of teeth. The anatomical tooth aspect of origin was occlusal/ incisal in 41.5% and crown in 82.9% of teeth. Moreover, 51.2% of GCs opened in palatal/lingual cortex and 63.4% of canals were not located along the tooth long axis. Finally, GC was detected in 85.7% of teeth undergoing the crown formation stage.

**Conclusion:**

Although GC was introduced as an eruption pathway, this canal is also present in impacted teeth. This means that presence of this canal does not promise the normal eruption of tooth and the anatomical characteristics of GC may influence the eruption process.

## Introduction

Tooth eruption is a physiological process involving a variety of anatomical components. Root development, alveolar bone growth, pulp development, and a mix of follicular theory and genetic factors, which is more widely acknowledged nowadays, are all explanations for this process [ 1 - 2 ]. The follicular theory postulates that dental follicles play an important role to guide the tooth and inducing bone resorption to create an eruption pathway during the intrabony phase [ 1 - 3 ]. Gubernacular canal (GC) and gubernacular cord were first introduced by John Hunter [ 4 ]. This canal originates from the follicle of permanent teeth and extends to the alveolar crest palatal/ lingual to deciduous teeth. It is filled with a gubernacular cord, which includes peripheral nerves, blood, and lymphatic vessels, as well as remnant epithelial cells of the dental lamina [ 2 ]. The gubernacular cord also contains many chemical mediators, including the epidermal growth factor (EGF), which has the ability to activate osteoclasts [ 2 ]. As a result, bone resorption occurs via this canal, whereby an eruptive pathway from the dental follicle to the gingiva is created [ 2 , 5 - 6 ]. Although it was suggested that GC acts as a guide for tooth eruption, its presence was proved only in permanent successional teeth and those without deciduous predecessor, but its presence has not been definitely determined in primary teeth [ 5 , 7 - 8 ]. However, it was stated that the absence of GC may indicate failure in tooth eruption [ 5 ], but this structure is observed in teeth with eruption failure too [ 9 ]. This makes the relationship between the failure of tooth eruption and GC characteristics unclear. Some studies [ 10 - 11 ] suggested that the gubernacular cord may play a role in the formation of adenomatoid odontogenic tumor (AOT) and ameloblastoma, since these tumors are composed of dental lamina remnants. The results of previous studies also have indicated a significant relationship between odontoma and the presence of GC [ 10 - 11 ].

On radiographic images, GC is described as a radiolucent/ hypodense canal with corticated boundaries connected to the dental follicle [ 7 ]. The delicate anatomic structure of GC, besides inherent superimpositions on two-dimensional radiographies makes its visualization on periapical and panoramic images difficult. GC can be mistaken for the nutrient canal, an accessory branch of the inferior alveolar canal, or even the fistular tract on radiographs regarding its position in the jaw. Nowadays, with the extensive use of cone beam computed tomography (CBCT) in dentistry, GC is detected more frequently by clinicians, and questions about its role in tooth development and eruption were raised. However, few clinical studies have focused on the presence, appearance, and importance of these structures. Hence, this study was designed to investigate the present rate of GC in impacted teeth (teeth that have failed to erupt at expected time) as well as anatomical characteristics of GC, which may be related to the eruption failure that occur in fully impacted teeth in alveolar bone by employing CBCT images in a population of south Iran.

## Materials and Method

**Figure 1:**
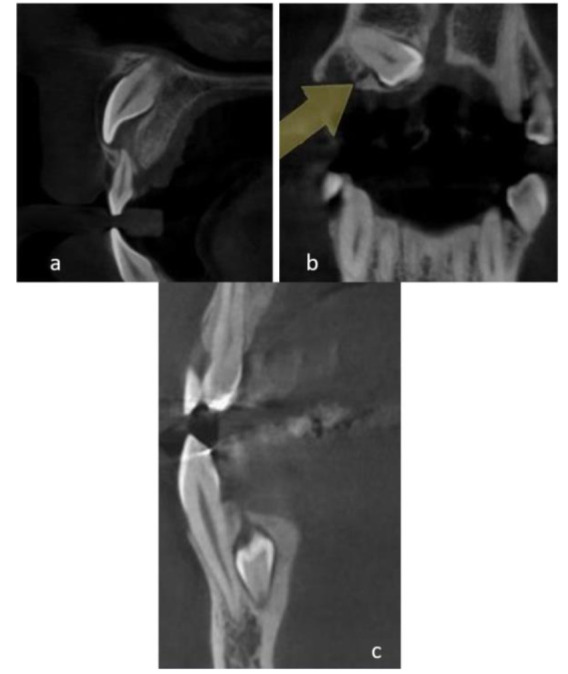
The origin of canal from the anatomical surface of tooth (**a:** buccal, palatal/lingual, **b:** mesial/distal and **c:** occlusal/ incisal)

**Figure 2:**
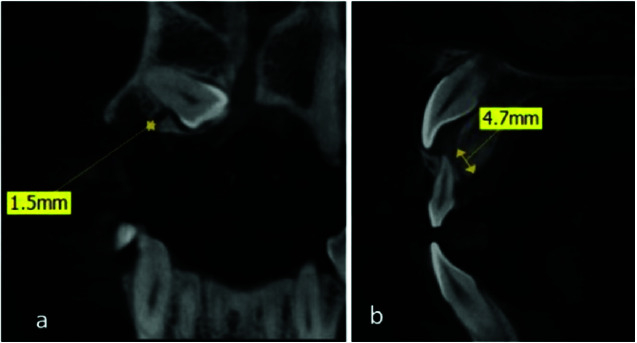
Measurement of the **a:** canal length and **b:** width

Fifty CBCT images of 77 impacted teeth were obtained from patients referring to a private maxillofacial radiology center in Bandar Abbas, Iran from January 2017 to March 2018. CBCT machine was a Pax Duo3D–Vatech/Korea with the field of view 5×5cm, 5×8cm and 8.5× 8.5cm, voxel sizes of 0.12 and 0.2mm and exposure factors of 80-90 KVp and 5-7mA. CBCT images were analyzed by EZ3D software. Inclusion criteria were impacted supernumerary and permanent teeth. The exclusion criteria were third molar teeth, positive history of surgery or pathologic conditions in the site of examined tooth, poor quality CBCT examinations with severe metal and motion artifacts and impacted teeth with dehiscent or fenestrated bony coverage. A maxillofacial radiologist with more than eight years of expertise assessed the pictures. On CBCT, GC is seen as a hypodense band with cortical borders connected to a dental follicle of an impacted tooth. CBCT images were examined in axial, sagittal, coronal, and oblique planes to determine the presence of GC, the anatomical tooth aspect which GC attached to (buccal, palatal/lingual, mesial, distal, and occlusal/incisal) (Figure 1). Moreover, the attachment site of GC to the crown, root or cervical part of the tooth, the direction of GC toward the adjacent cortical plate of alveolar bone (buccal cortex, lingual/ palatal cortex, and alveolar crest), the direction of GC in relation to the tooth long axis (along the long axis or not) and length and width of the GC (Figure 2) were studied.

The frequency of the GC in relation to tooth type (incisor, premolar and molar), development stage of the examined tooth (crown or root formation), and tooth position (normal, horizontal, angular, and inverted) were also analyzed.

## Results

CBCT images from 50 patients (29 female and 21 male) with 115 impacted teeth were evaluated. Of these, 38 teeth were excluded due to the lack of bony coverage. From totally 77 evaluated teeth (58 maxillary and 19 mandibular teeth), 41 (53.2%) had GC and 36 teeth (46.8%) did not. Among 58 maxillary teeth, 32 teeth (55.2%) had the GC and 26 teeth (44.8%) had not. From 19 mandibular teeth, 9 teeth (47.4%) had GC and 10 teeth (52.6%) did not. There was no statistically significant relationship between GC detection frequency and upper or lower jaw (p= 0.554). The distribution of different tooth types with and without GC is presented in Table 1. Among 41 teeth with GC, the tooth aspect of GC origin was buccal in 1 tooth, palatal/lingual in 7 teeth, occlusal/incisal in 17 teeth, mesial in 3 teeth, and distal in 13 teeth with a significant difference (*p*< 0.001). The distribution of GC location in different parts of tooth was detected as crown in 34 teeth (82.9%) and crown-root interface (tooth cervix) in 7 teeth (17.1%). The prevalence of crown location for GC was higher with a significant difference (*p*< 0.001). In 15 teeth (36.6 %), the detected GC was along tooth long axis while in the remaining 26 teeth (63.4%) it was not. Table 2 reports the distribution of adjacent cortical plates to which GCs open. The results indicate that GCs open to the palatal/lingual cortex with a significantly higher prevalence than buccal and alveolar crest cortices (*p*= 0.004).

**Table 1:** Distribution of different tooth types with and without gubernacular canal (GC)

Total	Supernumerary Tooth		2nd molar		2nd Premolar		1st Premolar		Canine		Central	
	WOGC *	WGC **	WOGC	WGC	WOGC	WGC	WOGC	WGC	WOGC	WGC	WOGC**	WGC*
77	6	15	0	1	3	4	0	1	19	25	3	0

* With GC

** Without GC

**Table 2:** Distribution of adjacent cortical plates as gubernacular canal opening site

0 (0.0%)	Buccal Cortex
21 (51.2%)	Palatal/ Lingual Cortex
4 (9.8%)	Alveolar crest Cortex
16 (39%)	None

The minimum, maximum and mean GC lengths were 1, 5 and 3.04 mm, respectively (SD= 3.04). The minimum, maximum, and mean canal widths were 1, 2.80 and 1.60mm, respectively (SD= 0.45).Among 14 teeth in the crown development stage, 12 (85.7%) teeth had GC. On the other hand, out of 63 teeth in the root development stage, 29 (46%) teeth had GC, where the difference was statistically significant (*p*= 0.007). The results of GC detection in teeth considering the different tooth positions as normal, oblique or angular, horizontal, and inverted are summarized in Table 3. Statistically, there was no correlation between tooth position and presence of GC (*p*= 0.114 from fisher’s exact test).

**Table 3:** Relationship between the gubernacular canal (GC) presence and the tooth position

Inverted	Horizontal	Oblique	Normal	Tooth Position
2(100%)	4(66.7%)	31(56.4%)	4(28.6%)	WGC^*^
0	2(33.3%)	24(43.6%)	10(71.4%)	WOGC^**^

## Discussion

GC is a canal that originates from the follicles of unerupted permanent teeth to the outer palatal/ lingual cortex of the alveolar process. The canal is filled with a gubernacular cord and consists of peripheral nerves, blood, lymphatic vessels, as well as epithelial cells formed from remnants of the dental lamina [ 2 , 6 ]. During tooth eruption, the canal significantly widens through which the tooth passes, and when the dental follicle joins the alveolar crest, GC disappears [ 12 ]. Oda *et al*. [ 13 ] stated that about 80% of odontomas have GTs, which indicates that the presence or absence of GTs could be useful for a differential diagnosis between complex odontomas and other jaw lesions with high-density structures. They found intact or wide GCs at the top of nearly 90% of odontogenic tumors or cysts on CT, indicating that the presence or absence of GTs in masses might be a highly valuable finding for distinguishing between odontogenic and non-odontogenic tumors or cysts [ 13 ]. The present study, performed only on impacted teeth, found a GC detection rate of 53.2%. Gaêta-Araujo *et al*. [ 7 ] showed observed GC detection rates of 87.1%, 62.9%, and 94. 1% in impacted teeth, teeth with delayed eruption, and teeth with normal eruption, respectively. Oda *et al*. [ 9 ] reported a detection rate of 90% in teeth with normal eruption and a lower GC prevalence rate in the teeth with delayed eruption (central incisor: 81.1%, lateral incisor: 83.3%, canine: 50%). The detection rate of GC in mesiodens was reported to be significantly lower (23.4%).

Nishida *et al*. [ 5 ] also reported that the GC detection rate in mesiodens teeth was far lower than in other teeth (1.6%). Although GC had previously been thought to be an eruption channel, new findings reveal that this canal is also seen in the impacted teeth, suggesting that the presence of this canal does not guarantee tooth eruption [ 5 ]. There was no significant association between GC prevalence and jaw and tooth type in this investigation. The current study revealed that the presence of GC is not limited only to the teeth with deciduous precursors; rather, there were also molar and supernumerary teeth with the GC. Consistent with the current study, Nishida *et al*. [ 5 ] and Ahmed *et al*. [ 6 ] reported the presence of GC in molar teeth. However, Hodson [ 8 ] believe that the GC is only present in teeth that erupt after the deciduous teeth were exfoliated .Gaêta-Araujo *et al*. [ 7 ] found that GCs extended more commonly in the occlusal/incisal direction in both normal and aberrant erupting teeth, however additional directions (mesial, distal, buccal, and palatal/lingual) were frequently detected in teeth with abnormal eruption. In the present study on impacted teeth, the extension of GC was seen more frequently in directions other than occlusal/incisal (58.5%). It may result in GC in a developing tooth extending in occlusal/ incisal direction; it has more chance to erupt normally. It can be anticipated that if the GC in a developing tooth extend in occlusal/ incisal direction, the tooth has more chance to erupt normally. In the present study, which was performed on impacted teeth, the location of the GC was in the crown in 34 teeth. In the remaining 7 teeth with GC, its location was in the tooth cervix. Gaêta-Araujo *et al*. [ 7 ] classified the canal location in relation to the tooth structure as occlusal/incisal, middle of the crown, cervical, and root. The results of their study revealed that in 35 out of 144 teeth with abnormal eruption, the canal was located in the middle of the crown, cervix, and root. On the other hand, the GC had an occlusal/incisal location in 396 teeth with normal eruption. In a study on the GC characteristics in mesiodenses, Oda *et al*. [ 9 ] showed that the GC was located in the cervical and root regions of mesiodenses with inversely crown-root position, while it was mainly located in the crown of mesiodenses with a normal position. The results of the Oda *et al*.'s study [ 9 ] showed that in central and lateral teeth with normal eruption, the angle of GC related to long tooth axis was significantly different from the same angle in the teeth with delayed eruption. The current study was performed only on impacted teeth, which the GCs were along long tooth axis in only 36.6% of teeth, which is in line with the results of previous studies. These findings suggest that the GC is not often located along the tooth's long axis in impacted teeth and teeth with delayed eruption. Hence, attention to the angle between the GC and tooth long axis can help clinicians predict the future eruption of the tooth and consider it in treatment planning. In the cases of the present study, the GC opened more commonly to the palatal/lingual cortex (51.2%), followed by the alveolar crest (9.8%). However, in 39% of the teeth, the canal opened to the incisive canal or the PDL space of adjacent teeth. Consistent with these results, Gaeta-Araujo *et al*. [ 7 ] showed that the canal opened to the lingual or palatal cortex in most cases, while in 1.3% of cases, the site of GC opening was not detectable. The mean length and width of GC in the present study were 3.04 and 1.6 mm, respectively. Similarly, Nishida *et al*. [ 5 ] reported a mean canal length and width of 3 and 1 mm, respectively. Oda *et al*. [ 9 ] indicated in their study that the canal length and width were related to the eruption stage, and the closer the tooth was to the alveolar crest, the shorter the canal length would be. Gaêta-Araujo *et al*. [ 7 ] showed that the canal length was longer only in mandibular premolar teeth with delayed eruption and there was no significant difference between other groups in terms of their canal length. However, the canal width was significantly larger in impacted teeth and teeth with delayed eruption. In the present study, the impacted teeth were divided into four groups in terms of position status i.e. normal, angular (oblique), and inverted positions. Although the GC was seen in fewer teeth with normal position, this difference was not statistically significant. Gaêta-Araujo *et al*. [ 7 ] showed that there was a weak relationship between the rates of canal detection and the angle of tooth position in teeth with normal eruption. On the other hand, in impacted teeth, the detection rate of the GC was higher in horizontally positioned teeth than those with angular and normal positions (horizontal: 100%, angular: 86.2%, normal: 83.1%). Finally, based on this study's results, GC was found in 85.7% of the teeth in the crown development stage and 46% of those in the root formation stage. Gaêta-Araujo *et al*. [ 7 ] also concluded that the GC detection rate was significantly higher in the impacted teeth during the initial stages of tooth formation (100%).

This research has several drawbacks, including as a limited sample size and the absence of a control group of teeth with normal eruption. One suggestion is to examine imaging properties of GCs in children of various ages for better understanding the possible functions of GC in tooth growth and eruption.

## Conclusion

GC can be detected in impacted permanent and supernumerary teeth with a lower rate than normal erupting teeth. In most of these teeth, GC is not along the tooth long axis. Additionally, extension of GC in impacted teeth was seen more frequently in directions other than occlusal/incisal.

## Conflict of Interest

The authors declare that they have no conflict of interest.
